# Interleukin-1β Signaling in Dendritic Cells Induces Antiviral Interferon Responses

**DOI:** 10.1128/mBio.00342-18

**Published:** 2018-03-20

**Authors:** Lauren D. Aarreberg, Courtney Wilkins, Hilario J. Ramos, Richard Green, Michael A. Davis, Kwan Chow, Michael Gale

**Affiliations:** a Center for Innate Immunity and Immune Disease, Department of Immunology, University of Washington School of Medicine, Seattle, Washington, USA; St. Jude Children's Research Hospital

**Keywords:** IL-1, West Nile virus, flavivirus, genomics, inflammasome, innate immunity, interferon, virus

## Abstract

Induction of interferon beta (IFN-β), IFN-stimulated genes (ISGs), and inflammatory responses is critical for control of viral infection. We recently identified an essential linkage of stimulation of the inflammatory cytokine interleukin-1β (IL-1β) and induction of ISGs that function as host restriction pathways against the emerging flavivirus West Nile virus (WNV) *in vivo*. Here we utilized *ex vivo* global transcriptome analysis of primary dendritic cells, known targets of WNV replication, to define gene signatures required for this IL-1β-driven antiviral response. Dendritic cells that were deficient in IL-1 receptor signaling showed dysregulation of cell-intrinsic defense genes and loss of viral control during WNV infection. Surprisingly, we found that in wild-type cells, IL-1β treatment, in the absence of infection, drove the transcription of IFN-β and ISGs at late times following treatment. Expression of these antiviral innate immune genes was dependent on the transcription factor IFN regulatory factor 3 (IRF3) and appears to reflect a general shift in IL-1β signaling from an early inflammatory response to a late IFN-mediated response. These data demonstrate that inflammatory and antiviral signals integrate to control viral infection in myeloid cells through a process of IL-1β-to-IRF3 signaling crosstalk. Strategies to exploit these cytokines in the activation of host defense programs should be investigated as novel therapeutic approaches against individual pathogens.

## IMPORTANCE

West Nile virus is an emerging mosquito-borne flavivirus that can result in serious illness, neuropathology, and death in infected individuals. Currently, there are no vaccines or therapies for human use against West Nile virus. Immune control of West Nile virus infection requires inflammatory and antiviral responses, though the effect that each arm of this response has on the other is unclear. The significance of our research is in defining how virus-induced inflammatory responses regulate critical antiviral immune programs for effective control of West Nile virus infection. These data identify essential mechanisms of immune control that can inform therapeutic efforts against West Nile virus, with potential efficacy against other neuroinvasive viruses.

## INTRODUCTION

Virus infection initiates innate immune and inflammatory responses that function to restrict viral replication and spread while serving to modulate the adaptive immune response for effective viral clearance. Type I interferon (IFN) and interleukin-1β (IL-1β) are central mediators driving innate antiviral immunity and inflammation, respectively (1–4). Though both cytokines are typically induced during acute virus infection, the temporal nature of their induction over the course of a specific virus infection and how each cytokine influences the actions of the other to drive downstream gene expression are not well understood. Evidence for positive and negative coregulation of each can be found in pathogen- and cell-specific contexts (5). Several studies have demonstrated that both IFN and IL-1β are critical cytokines for defense against West Nile virus (WNV) with distinct and concerted roles in directing host immunity (6–9).

WNV is a member of the single-stranded RNA virus family *Flaviviridae*. Over the past 18 years, WNV has emerged in North America and continues to cause infection and disease (10, 11). While the virus is normally maintained between mosquito and avian reservoirs, incidental infection of humans occurs through the bite of infected mosquitoes (12, 13). WNV initially replicates at the site of infection before spreading to the draining lymph nodes and spleen, where it replicates in subsets of macrophages and dendritic cells (DCs) (14). WNV is neurotropic, and although the virus is usually controlled in the periphery, it can spread to the central nervous system (CNS), where infection of neurons and induction of inflammation can lead to encephalitis and death (14–16). While inflammatory cell recruitment and function are necessary for limiting WNV pathogenesis, inflammation must be tightly controlled to prevent inflammation-mediated destruction of CNS tissue and disease (1, 17–19).

The type I IFN response is a major component of antiviral innate immunity. Induction of IFN-β is triggered downstream of pattern recognition receptors (PRRs), including the RIG-I-like receptors (RLRs) and Toll-like receptors (TLRs) (20, 21). PRRs recognize components of the virus and signal through conserved pathways to activate transcription factors belonging to the NF-κB and IFN regulatory factor (IRF) families to induce IFN-β expression (13, 22, 23). IFN-β is secreted from the cell and acts in autocrine and paracrine manners through the ubiquitous IFN-α/β receptor (IFNAR) to activate its receptor-associated kinases. These kinases can, in turn, phosphorylate and activate signal transducer and activator of transcription 1 (STAT1) and STAT2 for the assembly of the IFN-stimulated gene factor 3 (ISGF3) complex, which acts to induce the transcription of hundreds of ISGs that include known antiviral effector molecules (21). Components of the RLR signaling pathway are absolutely required for host clearance of WNV, as mice deficient in RIG-I, MDA5, MAVS, or IFN-β are unable to control WNV infection and are highly susceptible to WNV-induced death (7, 9, 24, 25).

IL-1β is one of a family of cytokines that includes IL-1α, IL-18, and IL-33 (1, 3). Its primary receptor, the IL-1 receptor (IL-1R), is homologous to the TLRs in its downstream signaling components and is constitutively expressed in most cell types (26). IL-1β signals through IL-1R to activate MyD88 and NF-κB and drive the expression of genes required for immune-mediated inflammation, effective adaptive immunity, and antiviral control (26–28). IL-1β induction and secretion are stimulated by a number of viruses, including influenza A virus, herpes simplex virus, Sendai virus, vesicular stomatitis virus, hepatitis C virus, dengue virus, and St. Louis encephalitis virus (28–30). Additionally, IL-1β-regulated inflammation of the brain is required for clearance of neurotropic viruses, including WNV and Japanese encephalitis virus (6, 31, 32).

Inflammatory molecules such as IL-1β and type I IFN are generally considered to be mutually antagonistic (5). IFN-β regulates inflammatory homeostasis by decreasing IL-1β production and inflammasome-mediated IL-1β processing, thereby preventing uncontrolled tissue destruction by inflammatory cytokines (33–35). IRF3 was shown to suppress the expression of proinflammatory genes such as those for IL-1 and tumor necrosis factor alpha (TNF-α) in microglia (36), while IL-1β was conversely found to decrease the ability of IRF3 to accumulate in the nucleus and bind to the IFN-sensitive response element (ISRE) in liver cells (37). Additionally, IL-1β-induced eicosanoids were found to limit type I IFN production in an *in vivo* model of *Mycobacterium tuberculosis* infection (38). However, the cross-regulation of inflammatory and IFN responses is not entirely antagonistic, as mice defective in IL-1R or IFNAR show defects in both responses (8, 39, 40).

IL-1β induction through the NLRP3 inflammasome was recently identified as a key component of host immunity to WNV infection (6, 8). WNV infection induced the acute production of IL-1β both *in vivo* and in *ex vivo* cortical neuron isolates. Loss of IL-1β signaling in IL-1R-deficient (*Il-1r*^*−/−*^) mice led to enhanced accumulation of WNV in the CNS but not the periphery of infected mice, resulting in increased pathogenesis and mortality rates (8). Importantly, we found that type I IFN levels were reduced in the draining lymph nodes and delayed in the CNS of WNV-infected mice in the absence of IL-1R signaling. Additionally, IL-1β and IFN-β acted synergistically to control WNV in *ex vivo* cultures of cortical neurons, suggesting cross-regulation of these cytokines that is required for effective antiviral control (8). As it has been suggested that myeloid cells promote WNV entry into the CNS via a “Trojan horse” mechanism (14), it is likely that the defect in viral control in *Il-1r*^*−/−*^ mice may be partially due to the reduced IFN levels in the draining lymph nodes, allowing for decreased control of virus in macrophages and DCs that go on to infiltrate the CNS and enhance encephalitic disease.

In this study, we address the role of IL-1R signaling in infection of primary macrophages and DCs, known target cells of WNV infection. We demonstrate that *ex vivo* cultures of *Il-1r*^*−/−*^ macrophages and DCs are unable to fully control WNV at late times postinfection and that this lack of antiviral control is associated with a loss of effective type I IFN responses in these cells. Significantly, we show that IL-1β treatment of bone marrow-derived DCs (BMDCs) results in induction of IFN-β and ISGs at late time points posttreatment and in the absence of infection. Our data suggest that the cross-regulation of IL-1β and IFN-β is required to effectively clear WNV infection.

## RESULTS

### IL-1β signaling is required for control of WNV infection in myeloid cells.

**FIG 1:**
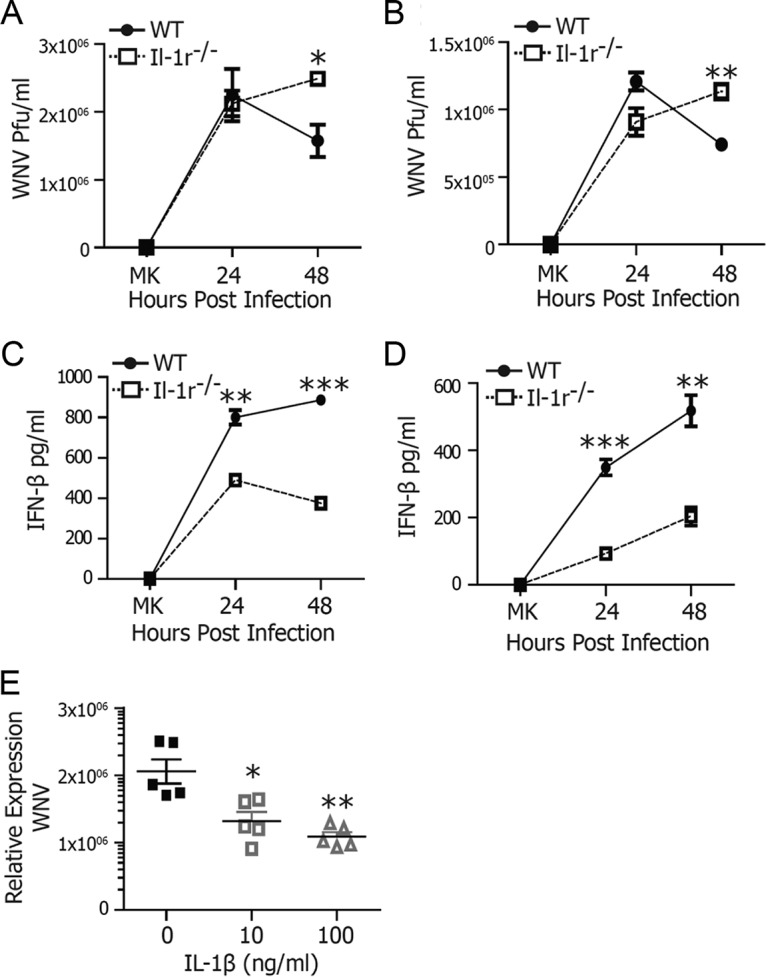
IL-1 signaling is required for WNV control. BMDCs (A, C) or BMMs (B, D) from WT or *Il-1r*^*−/−*^ mice were infected with WNV at an MOI of 2.5 and compared with mock-infected cells. At 24 and 48 h, WNV titers were determined by plaque assay (A, B) and IFN-β levels were measured by ELISA (C, D). (E) IL-1β (0, 10, or 100 ng/ml) was titrated onto WT BMDCs 24 h prior to infection with WNV at an MOI of 2.5. WNV RNA was measured by qRT-PCR at 48 h p.i. The data are averages of three (A to D) or five (E) independent experiments. Asterisks indicate values that are statistically significantly different by Mann-Whitney U test (A, B) or by unpaired *t* test (C to E) (*, *P* < 0.05; **, *P* < 0.01; ***, *P* < 0.001). MK, mock treatment.

Induction of type I IFN and the programing of an antiviral ISG response are critical for control of WNV replication (7, 9, 24, 25). Recently, we identified NLRP3 inflammasome activation and IL-1β signaling as key host restriction pathways important in the maintenance of optimal IFN and ISG responses to control WNV replication in neurons and the infected CNS (8). In contrast to neurons, which are highly permissive to WNV replication, myeloid cells can control WNV replication in a type I IFN-dependent manner (7). Therefore, to understand the mechanism by which IL-1β regulates antiviral control of WNV, we examined a requirement for this pathway in the control of WNV in primary myeloid cells. BMDCs and bone marrow-derived macrophages (BMMs) from wild-type (WT) and IL-1R-deficient (*Il-1r*^*−/−*^) mice were prepared and challenged with WNV. WNV replicated to similar titers in both WT and *Il-1r*^*−/−*^ BMDCs (Fig. 1A) and BMMs (Fig. 1B) at 24 h postinfection (p.i.). However, while WT cells controlled WNV by 48 h p.i., *Il-1r*^*−/−*^ cells showed increased viral replication and lack of viral control at this time (Fig. 1A and B).

The lack of viral control in IL-1R signaling-deficient cells suggested a similar defect in cell-intrinsic immunity to the virus as we previously observed in neurons (8). Therefore, we next examined type I IFN production in BMDCs and BMMs after a WNV challenge. In accordance with the lack of viral control, *Il-1r*^*−/−*^ BMDCs and BMMs displayed reduced IFN-β secretion (Fig. 1C and D). These data further identify IL-1β as a key host restriction factor involved in the regulation of antiviral immunity by the modulation of type I IFN responses.

To determine if IL-1β exposure was sufficient to mediate antiviral activity in myeloid cells, we prepared BMDCs from WT animals and pretreated them with 0, 10, or 100 ng/ml IL-1β. After 24 h, cells were either challenged with WNV or left as uninfected controls. IL-1β treatment reduced WNV RNA levels by 2- to 5-fold compared to those in untreated cells (Fig. 1E). Virus reduction was comparable to levels of inhibition observed in neurons, suggesting a global contribution of IL-1β to the elicitation of immunity to WNV (8).

### IL-1β drives antiviral gene signatures in DCs.

**FIG 2:**
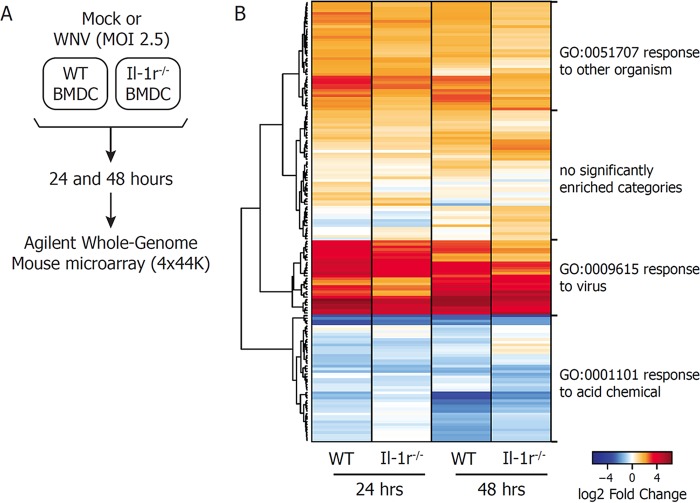
Genome-wide expression analysis of IL-1R-regulated genes. (A) Schematic diagram of the microarray design used in this study. WT or *Il*-*1r*^−/−^ BMDCs were mock infected or infected with WNV at an MOI of 2.5. Total RNA was extracted at 24 and 48 h p.i. and subjected to Agilent Whole Mouse Genome Microarray analysis. (B) Gene expression levels were determined as fold changes with respect to matched, mock-treated controls. A significant change is defined as a >1.5-fold increase or decrease with respect to mock treatment, with a BH-adjusted *P* value of <0.05. IL-1R-regulated genes were defined as those whose fold changes with respect to mock treatment in *Il*-*1r*^−/−^ BMDCs were >1.5-fold decreases compared with WT cells, with a BH-adjusted *P* value of <0.05. WNV-induced expression of IL-1R-regulated genes was plotted on a heat map with hierarchical clustering by Euclidean distance. Gene clusters are labeled with the most significantly enriched biological process in that group.

To examine the mechanism by which IL-1β participated in the control of WNV infection, we utilized global transcriptome analysis of BMDCs to define the gene signature associated with WNV infection and host defense induction (Fig. 2A). BMDCs were prepared from WT or *Il-1r*^*−/−*^ mice. Cells from mice of both genotypes were infected with WNV or left untreated as time-matched, mock-treated controls. Total RNA was harvested at 24 and 48 h p.i., and relative gene expression levels were determined by Agilent Whole Mouse Genome Microarray analysis (4×44K chip). Significant up- or downregulation of the expression of genes with respect to that in mock-treated controls was defined as a >1.5-fold change in expression, with a Benjamini-Hochberg (BH)-corrected *P* value of <0.05 (see Table S1 in the supplemental material). Gene expression patterns driven by WNV infection of WT and *Il-1r*^*−/−*^ BMDCs were then compared to define genes whose expression is regulated by IL-1R signaling. Gene expression changes that differed significantly between the two genotypes (as defined by the statistical criteria described above) were visualized by heat map for both 24- and 48-h samples (Fig. 2B). Interestingly, genes dysregulated in *Il-1r*^*−/−*^ BMDCs are involved in the response to viruses and the response to other organisms (as determined by Enrichr analysis of Gene Ontology biological processes [41]), indicating loss of antiviral control in the absence of IL-1R signaling (Fig. S1; Table S2). These data demonstrate that IL-1β signaling regulates innate immune response genes during WNV infection of DCs.

10.1128/mBio.00342-18.1 FIG S1  IL-1R signaling is necessary for induction of antiviral response genes. Antiviral response genes whose expression is more up- or downregulated during WNV infection in WT BMDCs as determined by a >1.5-fold increase or decrease with respect to IL-1R knockout BMDCs, with a BH-corrected *P* value of <0.05, in a microarray analysis. IL-1R-regulated genes were plotted on a heat map with hierarchical clustering by Euclidean distance. Download FIG S1, PDF file, 0.3 MB. Copyright © 2018 Aarreberg et al. 2018 Aarreberg et al. This content is distributed under the terms of the Creative Commons Attribution 4.0 International license.

10.1128/mBio.00342-18.4 TABLE S1  Genome-wide expression analysis of IL-1R-regulated genes. WT or *Il*-*1r*^−/−^ mutant BMDCs were mock infected or infected with WNV at an MOI of 2.5. Total RNA was extracted at 24 and 48 h p.i. and subjected to Agilent Whole Mouse Genome Microarray analysis. Gene expression levels were determined as fold changes with respect to matched, mock-treated controls. A significant change is defined as a >1.5-fold increase or decrease with respect to mock treatment, with a BH-adjusted *P* value of <0.05. IL-1R-regulated genes were defined as those whose fold changes with respect to mock treatment in *Il*-*1r*^−/−^ BMDCs were >1.5-fold decreases with respect to WT cells, with a BH-adjusted *P* value of <0.05. Download TABLE S1, XLSX file, 0.3 MB. Copyright © 2018 Aarreberg et al. 2018 Aarreberg et al. This content is distributed under the terms of the Creative Commons Attribution 4.0 International license.

10.1128/mBio.00342-18.5 TABLE S2  IL-1R-regulated antiviral response genes. Antiviral response genes whose expression is more up- or downregulated during WNV infection in WT BMDCs as determined by a >1.5-fold increase or decrease with respect to IL-1R knockout BMDCs, with a BH-corrected *P* value of <0.05, in a microarray analysis (Enrichr analysis of Gene Ontology biological processes [GO:0051707, responses to other organisms] and [GO:0009615, responses to viruses] [41]). Download TABLE S2, XLSX file, 0.01 MB. Copyright © 2018 Aarreberg et al. 2018 Aarreberg et al. This content is distributed under the terms of the Creative Commons Attribution 4.0 International license.

### IL-1β signaling enhances ISG responses after WNV infection.

**FIG 3:**
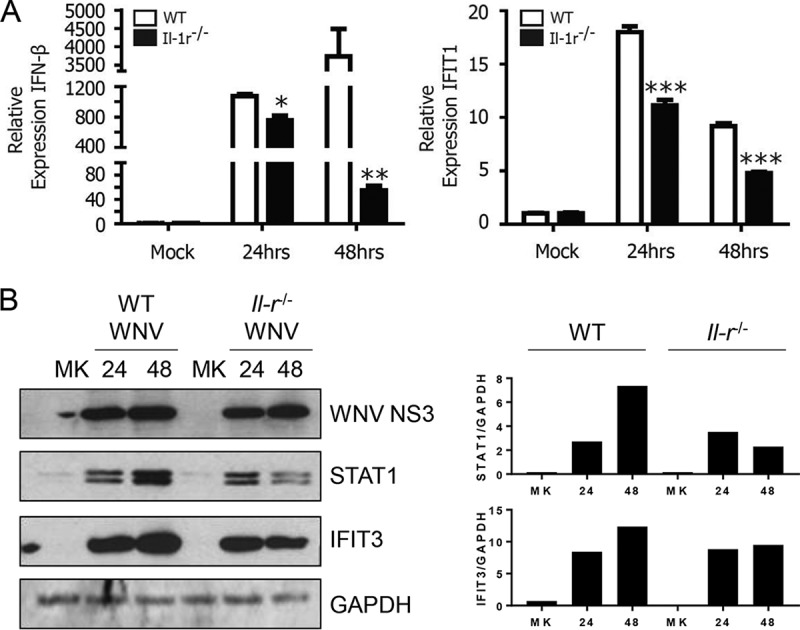
IL-1 signaling enhances antiviral responses. (A) WT or *Il-1r*^*−/−*^ BMDCs were mock infected or infected with WNV at an MOI of 2.5. Expression of IFN-β and IFIT1 was measured by qRT-PCR at 24 and 48 h p.i. relative to that in matched, mock-treated controls. (B) Total cell WNV NS3, STAT1, and IFIT3 protein levels were measured by immunoblotting with GAPDH as a loading control (left). Densitometry analyses of STAT1 and IFIT3 protein abundance were compared against GAPDH abundance for each condition (right). The data are the averages of three independent experiments. Asterisks indicate values that are statistically significantly different between WT and *Il*-*1r*^−/−^ cells by unpaired *t* test (*, *P* < 0.05; **, *P* < 0.01; ***, *P* < 0.001). MK, mock treatment.

To understand the effect of IL-1R signaling requirements on ISG induction following WNV infection, we examined ISG expression by quantitative real-time PCR (qRT-PCR) and immunoblotting. The WNV-driven expression of the gene for IFN-β, an IRF3 and IRF7 target, is slightly lower in *Il-1r*^*−/−*^ BMDCs at 24 h p.i. than that in WT BMDCs, but this difference in expression is exacerbated by 48 h (Fig. 3A, left). IFIT1 is regulated by both IRF3- and IFN-responsive promoter sites, and it shows depressed expression in the absence of IL-1R at both times by qRT-PCR (Fig. 3A, right) (42, 43). Additionally, expression of the ISG-encoded proteins STAT1 and IFIT3 is not maintained during WNV infection in the absence of IL-1R signaling (Fig. 3B). Together, these results confirm that ISG expression is negatively altered by the lack of IL-1β signaling in WNV-infected BMDCs. Moreover, curtailed expression of these genes appears to associate with the lack of control of WNV in *Il-1r*^*−/−*^ BMDCs at 48 h p.i. (see Fig. 1A).

### IL-1β drives the expression of IFN-β and ISGs in the absence of infection.

**FIG 4:**
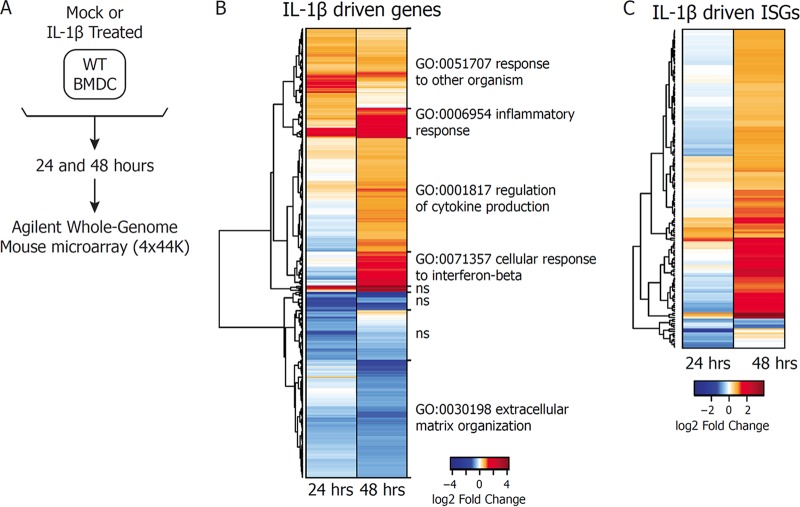
IL-1β treatment drives expression of IFN-β and ISGs. (A) Schematic diagram of the microarray design used in this study. WT BMDCs were mock treated or treated with IL-1β (100 ng/ml). Total RNA was extracted at 24 and 48 h posttreatment and subjected to Agilent Whole Mouse Genome Microarray analysis. (B) Gene expression levels were determined as fold changes with respect to matched, mock-treated controls. A significant changes is defined as a >1.5-fold increase or decrease with respect to mock treatment, with a BH-adjusted *P* value of <0.05. IL-1β-regulated genes were plotted on a heat map with hierarchical clustering by Euclidean distance. Gene clusters are labeled with the most significantly enriched biological process in that group. The abbreviation ns signifies no significantly enriched categories in that cluster. (C) IL-1β-driven genes were compared against genes found to be induced upon IFN-β treatment of WT BMDCs. ISGs regulated by IL-1β as defined for panel B were plotted on a heat map.

The surprising dysregulation of ISGs in *Il-1r*^*−/−*^ BMDCs during WNV infection led us to examine how IL-1R signaling affects gene expression in the absence of infection (Fig. 4A). As expected, treatment of WT BMDCs with IL-1β for 24 or 48 h resulted in an increase (upregulation) or decrease (downregulation) in the expression of a number of genes mapping to inflammatory responses (Fig. S2A) (41). An analysis of all of the genes whose expression is regulated by IL-1β treatment in WT BMDCs demonstrated that gene modules enriched in inflammatory response genes and genes involved in the response to other organisms are upregulated at both 24 and 48 h posttreatment, while genes involved in cytokine regulation and the cellular response to IFN-β are induced at the later time point (Fig. 4B; Table S3). To determine whether any of these innate immune genes were ISGs, we compared the list of IL-1β-driven genes to a published list of genes found to be induced following IFN-β treatment of WT BMDCs (44). We found that while a few ISGs were expressed at 24 h posttreatment, many more were driven by IL-1β at 48 h posttreatment (Fig. 4C; Table S4). Interestingly, a portion of the IL-1β-driven ISGs actually appeared to be downregulated at 24 h posttreatment but were then either back to the baseline or upregulated by 48 h posttreatment. qRT-PCR analysis confirmed that IFN-β and a number of ISGs were transcriptionally silent or even downregulated at 24 h after IL-1β treatment but were upregulated at 48 h following IL-1β treatment alone (Fig. S3). Consistent with these results, Gene Ontology analysis of biological processes upregulated following IL-1β treatment revealed an increased enrichment of genes involved in the response to viruses, as well as a loss of enrichment of type I IFN signaling pathways from the downregulated gene sets (Fig. S2B). These results demonstrate that IL-1β signaling in BMDCs leads to expression of ISGs in BMDCs at late times posttreatment.

10.1128/mBio.00342-18.2 FIG S2  IL-1β treatment drives inflammatory response genes. (A) Genes mapping to the Gene Ontology biological process term inflammatory response (GO:0006954) whose expression is up- or downregulated by IL-1β treatment at 24 or 48 h posttreatment as determined by a >1.5-fold increase or decrease with respect to matched, mock-treated cells, with a BH-corrected *P* value of <0.05, in a microarray analysis (41). (B) Genes upregulated at 24 and 48 h after IL-1β treatment were assessed for enrichment of Gene Ontology biological processes. Significant enrichment is defined as a BH-adjusted *P* value of <0.05. Enrichment (E) scores refer to the negative log of the adjusted *P* value. The enrichment score top five significantly enriched categories in each direction are plotted for 24 (top) or 48 (bottom) h of IL-1β treatment. Download FIG S2, PDF file, 0.4 MB. Copyright © 2018 Aarreberg et al. 2018 Aarreberg et al. This content is distributed under the terms of the Creative Commons Attribution 4.0 International license.

10.1128/mBio.00342-18.3 FIG S3  IL-1β treatment drives the expression of ISGs. WT BMDCs were mock treated or treated with IL-1β (100 ng/ml) for 24 or 48 h. Total RNA was harvested and subjected to qRT-PCR to determine relative levels of gene expression. The data are the averages of three independent experiments. Asterisks indicate values that are statistically significantly different between mock-treated controls and IL-1β-treated cells by unpaired *t* test (*, *P* < 0.05; **, *P* < 0.01; ***, *P* < 0.001). Download FIG S3, PDF file, 0.1 MB. Copyright © 2018 Aarreberg et al. 2018 Aarreberg et al. This content is distributed under the terms of the Creative Commons Attribution 4.0 International license.

10.1128/mBio.00342-18.6 TABLE S3  IL-1β-driven genes. WT BMDCs were mock treated or treated with IL-1β (100 ng/ml). Total RNA was extracted at 24 and 48 h posttreatment and subjected to Agilent Whole Mouse Genome Microarray analysis. Gene expression levels were determined by fold changes with respect to matched, mock-treated controls. A significant change is defined as a >1.5-fold increase or decrease with respect to mock treatment, with a BH-adjusted *P* value of <0.05. Download TABLE S3, XLSX file, 0.04 MB. Copyright © 2018 Aarreberg et al. 2018 Aarreberg et al. This content is distributed under the terms of the Creative Commons Attribution 4.0 International license.

10.1128/mBio.00342-18.7 TABLE S4  IL-1β-driven ISGs. IL-1β-driven genes from Table S3 were compared with genes found to be induced upon IFN-β treatment of WT BMDCs (44). Download TABLE S4, XLSX file, 0.01 MB. Copyright © 2018 Aarreberg et al. 2018 Aarreberg et al. This content is distributed under the terms of the Creative Commons Attribution 4.0 International license.

### Signaling requirements of IL-1β-driven responses.

**FIG 5:**
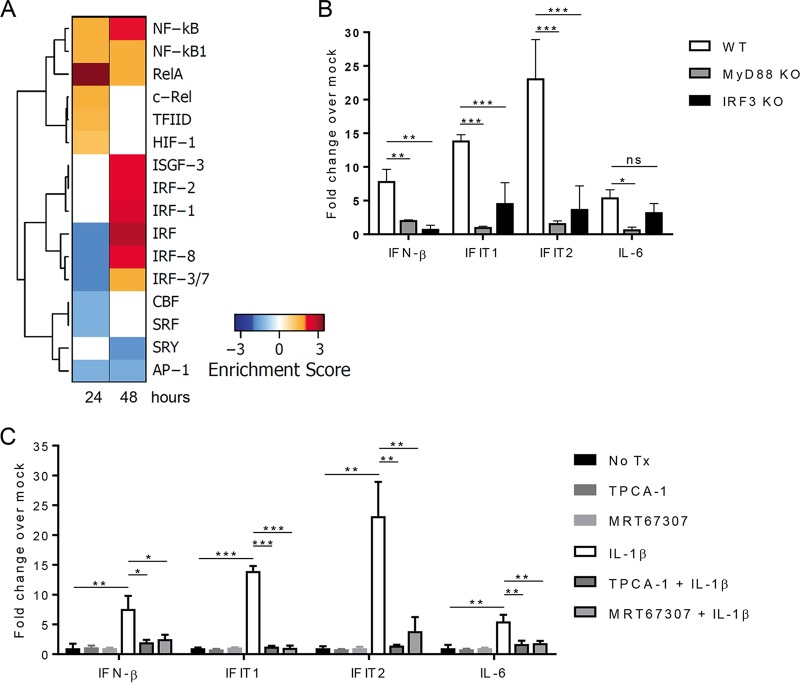
Signaling requirements of IL-1β-driven ISG responses. (A) Genes upregulated (red) or downregulated (blue) after 24 or 48 h of IL-1β treatment were assessed for enriched transcription factor binding sites (UCSC Genome Browser PWM in Enrichr [41, 74]). Significantly enriched sites are considered those with an adjusted *P* value of <0.05. Enrichment scores are defined as the negative log of the adjusted *P* value. (B) WT, *Myd88*^*−/−*^, and *Irf3*^*−/−*^ BMDCs were mock treated or treated with IL-1β (100 ng/ml) for 48 h. Gene expression levels were measured by qRT-PCR and are displayed relative to those of matched, mock-treated controls. (C) WT BMDCs were mock treated (No Tx) or pretreated with the IKKβ inhibitor TPCA-1 (50 nM) or the TBK1/IKKε inhibitor MRT67307 (2 µM) for 1 h and then mock treated or treated with IL-1β (100 ng/ml) for 48 h. The data are averages of three independent experiments and represent fold changes with respect to respective mock-treated controls. Asterisks indicate values that are statistically significantly different between WT and *Myd88*^*−/−*^ or WT and *Irf3*^*−/−*^ cells (B) or between treatment groups and mock-treated cells (C) by unpaired *t* test (*, *P* < 0.05; **, *P* < 0.01; ***, *P* < 0.001).

To identify the transcription factors linked with IL-1β signaling to drive ISGs and inflammatory molecules, we assessed the enrichment of promoter regions among lists of genes up- or downregulated following 24 or 48 h of IL-1β treatment (Fig. 5A). We found that the general IRF binding site and the IRF3/7 binding site are enriched within the list of downregulated genes at 24 h after IL-1β treatment but the IRF motifs are remarkably enriched in the list of genes upregulated at 48 h after IL-1β treatment, consistent with IL-1β driving a distinct crosstalk toward an innate immune antiviral response at 48 h posttreatment. The ISRE binding factor ISGF3 is also enriched at 48 but not 24 h posttreatment. Binding sites for the NF-κB family members cRel and RelA, as well as the general NF-κB binding site, are enriched at both 24 and 48 h after IL-1β treatment, although the enrichment pattern appears to be altered slightly at 48 h. This overall pattern is consistent with a shift in IL-1β signaling from an NF-κB-driven inflammatory response to an IRF-driven antiviral response.

To confirm the role of IRF signaling in ISG induction after IL-1β treatment, we treated BMDCs from WT or *Irf3*^*−/−*^ mutant mice with IL-1β and assessed the expression of genes identified in our transcriptomic analysis (Fig. 5B). While the induction of NF-κB-responsive IL-6 expression was not affected by the loss of IRF3, *Irf3*^*−/−*^ BMDCs were unable to express IFN-β. Similarly, the expression of ISGs IFIT1 and IFIT2 was largely reduced by IL-1β treatment of *Irf3*^*−/−*^ BMDCs compared to that in WT cells. These data demonstrate that the induction of ISGs by IL-1β is indeed through an IRF-dependent mechanism. NF-κB- and IRF-mediated transcriptional activity depends on their regulation by the canonical and noncanonical IκB kinases (IKKs) (45–47). The canonical IKKs IKKα and IKKβ activate NF-κB via phosphorylation and subsequent degradation of the NF-κB inhibitory molecule IκBα (45). The noncanonical IKKs include TBK1 and IKKε and are essential for the phosphorylation and activation of IRF3 (46, 47). Additionally, IKKε can regulate innate immune effector genes via modulation of STAT1 (48, 49). We examined the contribution of these kinases to IL-1β-induced gene expression through the use of a pharmacological inhibitor of the canonical IKKs (TPCA-1 [50]) or the noncanonical IKKs (MRT67307 [51]). Interestingly, inhibition of either the canonical or the noncanonical IKKs completely prevented IL-1β-induced expression of IFN-β, IFIT1, and IFIT2 (Fig. 5C). Additionally, both IKK families influence the expression of the NF-κB- and ISGF3-responsive gene for IL-6. As NF-κB is necessary for the induction of IFN-β in this context, inhibition of the canonical IKKs could affect secondary response genes downstream of IFN (i.e., ISGF3-driven genes) (52).

To define the signaling requirements of IL-1β-driven responses, we assessed whether the Toll-IL-1 receptor domain-containing adaptor protein MyD88 mediated this signature. WT and *Myd88*^*−/−*^ BMDCs were treated with IL-1β and gene expression was assessed by qRT-PCR. As expected, NF-κB- and IRF-mediated transcriptional changes induced by IL-1β are entirely dependent upon this essential signaling adapter (Fig. 5B). These results show that IL-1R/MyD88 signaling can activate both canonical and noncanonical IKKs to coordinately induce antiviral response genes through the actions of the NF-κB and IRF transcription factor families.

### Model of IL-1β signaling.

**FIG 6:**
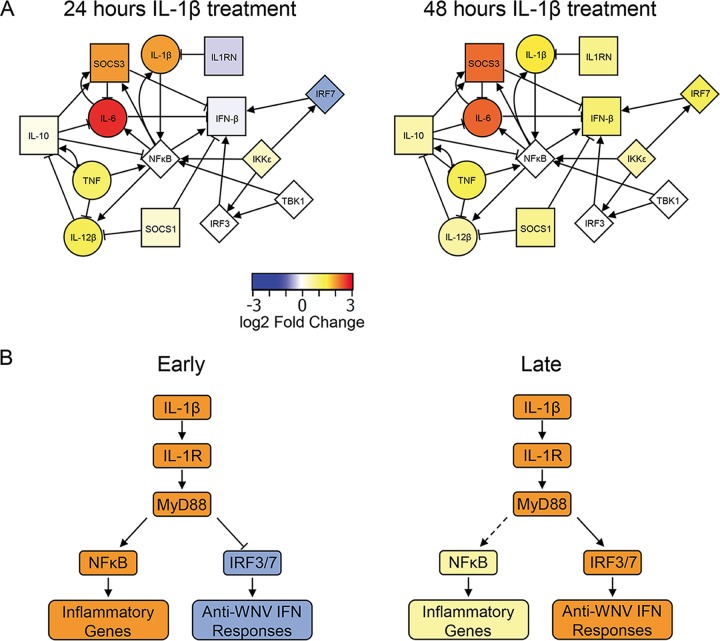
Model of IL-1β-driven ISG responses. (A) Network analysis of inflammatory and anti-inflammatory genes during IL-1β treatment. Nodes represent either genes induced by IL-1β treatment or signaling molecules and transcription factors regulating their expression. Circular nodes are considered inflammatory, whereas square nodes are considered anti-inflammatory. Diamond-shaped nodes represent signaling molecules and transcription factors involved in this network. Edges between nodes were curated from the InnateDB database (71) and represent either activation (arrows) or inhibition (bars). Node fill colors represent log_2_-fold changes in expression following IL-1β treatment with respect to mock-treated cells at the times indicated. (B) Model of IL-1β responses in BMDCs. At early times after IL-1β exposure, signaling to NF-κB leads to upregulation of inflammation-related genes while signaling to IRF3 and IRF7 is inhibited. At later times, the inflammatory response is dampened by IRF activation, leading to induction of an anti-inflammatory response. This anti-inflammatory response includes type I IFN and other antiviral genes that promote the maintenance of antiviral responses during WNV infection.

Finally, we used network analysis to examine the interplay between inflammatory and anti-inflammatory molecules following IL-1β treatment. As shown in Fig. 6A, we identified distinct regulatory nodes of IL-1β signaling according to our transcriptomic and kinase inhibitor data sets. At 24 h posttreatment, proinflammatory gene mRNA expression is high while the expression of antiviral ISGs like that for IRF7, a prominent biomarker of the antiviral/IFN response (53), is notably repressed. However, by 48 h posttreatment, the expression of inflammatory genes is either reduced or not substantially increased compared to that at 24 h. This change is concomitant with the upregulation of genes with known inhibitory functions toward inflammatory cytokines (26, 54, 55). At 48 h, IRF7 mRNA expression is induced, correlating with an increase in IRF3/7-responsive IFN-β and antiviral genes at later times after IL-1β exposure. Together, these data sets demonstrate a dynamic regulation of the IL-1β signaling outcomes for inflammatory and antiviral genes in a cell-intrinsic manner.

## DISCUSSION

Our study reveals that the loss of IL-1R has a detrimental effect on antiviral responses to WNV in BMDCs and macrophages, leading to reduced type I IFN and increased viral replication. Additionally, multiple functional classes of ISGs are disrupted in *Il-1r*^*−/−*^ cells in the induction and/or maintenance of expression throughout infection. Bioinformatic modeling suggests that the pattern of response to ISGs in the presence or absence of IL-1 signaling may be determined by the particular transcription factors responsible for gene expression levels and that the host transcription machinery is not optimally coordinated without some level of IL-1 signaling. Additionally, we found that IL-1β treatment of BMDCs led to early induction of proinflammatory genes but shifted at later times to the induction of anti-inflammatory genes that serve to dampen the inflammatory response following IL-1β treatment. Our previous study demonstrated that cortical neurons lacking IL-1R actually produce more IFN-β in response to WNV (8), suggesting that the specifics of cross-regulation between these pathways differs from that found in monocyte-derived cells.

Type I IFN and proinflammatory cytokines are each known to downregulate the production and function of the other (5), suggesting that the induction of IFN-β at late times after IL-1β treatment may serve as a mechanism to balance antimicrobial inflammatory function with pathological inflammation-mediated tissue damage. In our previous study, we found that subsets of microglia appear to become activated upon WNV entry into the CNS in infected mice (8). However, these microglia did not return to basal states at late times of infection in *Il-1r*^*−/−*^ mutant mice as they did in WT mice. This outcome implies a role for the IL-1 signaling pathway in the maintenance of homeostatic balance of inflammation in the CNS, particularly in macrophage- or DC-like cells. Consistent with this notion, IRF3 activation has been reported to act as a switch from proinflammatory “M1-like” to immunomodulatory “M2-like” phenotypes in microglia (36), and IFN has been reported to have a role in the homeostatic defense against IL-1-mediated inflammation and tissue damage (34).

Our data sets support a model of IL-1β-to-IRF3 crosstalk signaling in which at earlier times following IL-1β exposure of BMDCs, signaling through IL-1R and MyD88 to NF-κB leads to a canonical and well-described response of upregulation of inflammatory genes and cytokines to direct the classic inflammatory response to IL-1β. The opposing anti-inflammatory/antiviral response, including IRF3/7-mediated induction of IFN-β expression, is silent at these times after IL-1β exposure to allow for efficient inflammatory responses. At later times posttreatment, IL-1β continues to drive the expression of genes for inflammatory cytokines through NF-κB, albeit at lower levels than at earlier times posttreatment (Fig. 6B). However, by this time following IL-1β exposure, signaling has begun a regulatory anti-inflammatory response, including the expression of type I IFN and ISGs. Coordinate activation of NF-κB and IRF transcription factors results in the expression of critical antiviral genes. This dynamic crosstalk of IL-1β and IFN pathways may serve to both control inflammatory responses and sustain antiviral responses to WNV.

The crosstalk signaling by IL-1β to type I IFNs in cellular homeostasis is likely of particular importance beyond virus infection to impact autoimmune development and immune regulation. Depending on the particular autoimmune disease and stage of development, type I IFNs can promote disease through chemokine expression and antigen presentation or protect against damage through regulation of proinflammatory cytokines, including IL-1β and TNF-α (56). In clinical settings, inhibition of IL-1β through specific agonists or through IFN-β therapy is useful in limiting the development and progression of autoimmune and inflammation-mediated diseases, including rheumatoid arthritis and multiple sclerosis (18, 33, 57).

One of the best-studied scenarios of IL-1β cross-regulation with type I IFN is in the context of *M. tuberculosis* infection. IL-1β is absolutely required for effective host responses to *M. tuberculosis* infection (35). However, virulent strains of *M. tuberculosis* selectively trigger the induction of type I IFN, which inhibits the expression of protective IL-1β expression (58). Although this may also reflect an attempt by the host to limit inflammation-mediated tissue damage, *M. tuberculosis* is able to utilize the response to enhance its own infection and pathogenesis. This response is also relevant during viral infections in *M. tuberculosis*-infected patients, as type I IFN production during influenza virus infection exacerbates *M. tuberculosis* infection and disease progression (59). Conversely, IL-1β-induced eicosanoids were shown to inhibit the actions of type I IFN during influenza virus (60) or *M. tuberculosis* (38) infection, with opposite outcomes for disease. These studies highlight the complicated interplay between inflammation and IFNs during microbial infection.

Other groups have also observed connections between IL-1β signaling and IFN induction. IL-1β was found to activate IRF3 in cultured human fetal astrocytes that then induced the expression of IRF7 and IFN-β (61). The authors suggested that IL-1β produced by activated microglia may trigger IRF3 activity in astrocytes to amplify innate immune responses and provide a second line of defense against infection in the CNS. Additionally, TLR9-dependent activation of type I IFN and the anti-inflammatory cytokine IL-10 was found to be lacking in the absence of IL-1R, and BMDCs from *Il-1r*^*−/−*^ mice failed to mount protective type I IFN responses following TLR9 or TLR3 stimulation (62). Another group found that Huh7 hepatoma cells cotreated with IFN-α and IL-1β show potentiated ISG expression and phosphorylation of STAT1, while no ISG induction was observed upon the treatment of cells with IL-1β alone (40); these results suggest that the IFN–IL-1β combination might provide promoter enhanceosome activity through specific transcription factors that together drive enhanced ISG expression in these cells (63). IL-1R-mediated IFN production may not be limited to IL-1β, as IL-1α has also been shown to induce the transcription of IFN-β mRNA in human foreskin fibroblasts (64). These studies provide additional support for our finding that IL-1β signaling can be intricately linked to the induction of IFN-β and ISGs in a cell-specific manner.

There are several potential mechanisms by which IL-1β signaling may shift to induction of IFN-β at late times posttreatment. One such mechanism is shunting of signaling by the adapter molecule TNF receptor-associated factor 3 (TRAF3). TRAF3 is essential for the induction of type I IFNs and IL-10 in BMDMs but is dispensable for the expression of proinflammatory cytokines (65). TRAF3 must be ubiquitinated at residue K48 and subsequently degraded for MyD88-dependent TLR signaling to produce proinflammatory cytokines, while nondegradative K63-linked self-ubiquitination of TRAF3 leads to IFN-β induction (66). In other systems, IL-1 signaling has been shown to trigger the downregulation of deubiquitinating enzyme A (DUBA), which selectively cleaves K63-linked ubiquitin chains from TRAF3 to limit type I IFN responses (62). Although we have not detected this phenomenon in our system, the possibility remains that DUBA expression or function may be altered over the course of IL-1β treatment to manage the switch to anti-inflammatory gene induction. Another possible way in which IL-1β treatment may lead to IRF-dependent IFN-β expression is signaling through phosphatidylinositol 3-kinase (PI3K)/Akt. In microglia, overexpression of IRF3 via adenoviral vectors activated PI3K and Akt to induce the anti-inflammatory genes for IL-1RN, IL-10, and IFN-β (36). The authors suggested that Akt signaling may suppress miR-155 to modulate cytokine production. IFN and inflammatory cytokine signaling have, under other circumstances, been found to induce cellular microRNAs (miRNAs) that target components of IFN signaling (56), so modulation of miRNA expression and function is another mechanism by which IL-1β and IFN-β may cross-regulate each other. Certainly, there may be yet other mechanisms induced by IL-1β signaling that function to derepress IRF-mediated signaling at late times after exposure as a means of inflammatory resolution.

In summary, our studies demonstrate a cell-intrinsic cross-regulation of IL-1β signaling and type I IFN responses in myeloid cells that is required for optimal control of WNV infection. Further defining the mechanisms by which proinflammatory signaling switches to activate anti-inflammatory cytokines and antiviral ISG responses may reveal novel targets for the control of dysregulated immune responses in autoinflammatory disease, as well as in response to pathogens.

## MATERIALS AND METHODS

### Materials.

Recombinant murine IL-1β was purchased from Miltenyi Biotec, Inc.; reconstituted in sterile water; and stored at a concentration of 100 µg/ml at −20°C. TPCA-1 (Tocris) was reconstituted in ethanol and stored at 10 mM at −20°C. MRT67307 (Sigma) was reconstituted in sterile water and stored at a concentration of 15 mg/ml at −20°C. The working concentrations used are indicated in the figure legends.

### Viruses and cell lines.

WNV isolate TX 2002-HC (WNV-TX) titers were determined by a standard plaque assay on BHK-21 cells, and working stocks of WNV-TX were generated as previously described (9). BHK-21 cells were cultured in Dulbecco’s modified Eagle medium (DMEM) supplemented with 10% fetal bovine serum (FBS), HEPES, l-glutamine, sodium pyruvate, an antibiotic-antimycotic solution, and nonessential amino acids.

### Primary cell isolation and infection.

WT, IL-1R-deficient (*Il-1r*^*−/−*^), and MyD88-deficient (*Myd88*^*−/−*^) C57BL/6 mice were described previously (8). *Irf3*^*−/−*^ mice were a kind gift from T. Taniguchi. All mice were genotyped for positive identification and bred under specific-pathogen-free conditions in the animal facility at the University of Washington. Experiments were performed in accordance with University of Washington Institutional Animal Care and Use Committee guidelines. BMDCs were generated as follows. Cells were isolated from the bone marrow of WT, *Il-1r*^*−/−*^, *Myd88*^*−/−*^ or *Irf3*^*−/−*^ mice and cultured for 7 days in Roswell Park Memorial Institute (RPMI) 1640 medium supplemented with 10% FBS, l-glutamine, sodium pyruvate, an antibiotic-antimycotic solution, and nonessential amino acids in the presence of 20 ng/ml granulocyte-macrophage colony-stimulating factor and 20 ng/ml IL-4 (PeproTech, Rocky Hill, NJ). BMMs were generated as follows. Cells were isolated from the bone marrow of WT or *Il-1r*^*−/−*^ mice and cultured for 7 days in DMEM supplemented with 10% FBS, l-glutamine, sodium pyruvate, an antibiotic-antimycotic solution, and nonessential amino acids in the presence of 40 ng/ml macrophage colony-stimulating factor (PeproTech, Rocky Hill, NJ). BMDCs or BMMs (5 × 10^5^) were infected with WNV-TX at a multiplicity of infection (MOI) of 2.5 for 1 h, washed, and subsequently incubated for 24 or 48 h in the appropriate medium before downstream analyses.

### IFN-β ELISA.

For detection of IFN-β in cell culture supernatants, 100 µl of UV-inactivated supernatant was tested with mouse-specific enzyme-linked immunosorbent assay (ELISA) kits from PBL Biomedical Laboratories in accordance with the manufacturer’s protocol.

### Immunoblotting.

Protein extracts (20 µg) were analyzed by immunoblotting. The primary antibodies used to probe blots were goat anti-WNV NS3 (R&D Systems), rabbit anti-ISG49 (IFIT3; kindly provided by G. Sen), rabbit anti-GAPDH (FL-335; Santa Cruz), and rabbit anti-STAT1 (Cell Signaling) antibodies. The secondary antibodies used included peroxidase-conjugated goat anti-rabbit and donkey anti-goat antibodies (Jackson ImmunoResearch, Inc.). Densitometry analysis was performed with Image Studio Lite software (LI-COR).

### RNA extraction and analysis.

Total RNA was isolated from BMDCs with RNA extraction buffer (RLT; Qiagen) and the RNeasy kit in accordance with the manufacturer’s protocol (Qiagen). DNase-treated RNA (Qiagen) was then reverse transcribed to cDNA with a 1:1 mixture of random hexamers and oligo(dT) primers with the iScript Select cDNA synthesis kit (Bio-Rad). The WNV-specific RNA copy number was measured by single-step qRT-PCR by TaqMan technology via specific primer sets and probes as previously described (9). Gene expression was assessed by one-step SYBR green qRT-PCR with an ABI 7800 machine. The specific primer sets used for mouse glyceraldehyde-3-phosphate dehydrogenase (GAPDH), IFN-β, IL-1β, IL-6, Ms4a4b, Ms4a4c, Iigp1, and Tgtp1 are as follows: mGAPDH forward, CAACTACATGGTCTACATGTTC; mGAPDH reverse, CTCGCTCCTGGAAGATG; mIFNb forward, GGAGATGACGGAGAAGATGC; mIFNb reverse, CCCAGTGCTGGAGAAATTGT; mIL1b forward, ACGGACCCCAAAAGATGAAG; mIL1b reverse, CACGGGAAAGACACAGGTAG; mIL6 forward, GTTCTCTGGGAAATCGTGGA; mIL6 reverse, TGTACTCCAGGTAGCTATGG; mMs4a4b forward, TGCAGCAGGAGTGACACCTACAAA; mMs4a4b reverse, ACAGCCACACTGACTACACCCATT; mMs4a4c forward, CCTGTCAATTGCAGCAGGAGTGAA; mMs4a4c reverse, TGCAGCCAACACAGAGGTGATAGT; mIigp1 forward, AGTGTGCTCAATGTTGCTGTCACC; mIigp1 reverse, TTCATTCCCAATGCCTCTCAGGGT; mTgtp1 forward, TGCAAGTCTTACTGAGGCCACC; mTgtp1 reverse, ATGCTCCAGCCTTCATGGCTTCTA. mIFIT1 and mIFIT2 were purchased as premixed SuperArray primer sets (Qiagen).

### RNA preparation and oligonucleotide microarray processing.

Total RNA was harvested for array analysis with TRIzol LS. Samples were prepared and hybridized to Agilent Mouse Whole-Genome Oligo 4×44K Microarrays as previously described (67).

### Microarray analysis and bioinformatics.

Microarray data were analyzed with the R statistical programing language and Bioconductor (68, 69). Raw data were quantile normalized and then used for linear modeling with the limma package (70). Genes with significant changes following WNV infection or IL-1β treatment were defined as those with a >1.5-fold increase or decrease with respect to genotype- and time-matched controls, with a BH-corrected *P* value of <0.05. WT and *Il-1r*^*−/−*^ WNV responses with respect to those obtained with mock treatment were quantitatively compared by using the limma package and the criteria described above. Network analysis was run by manual curation with the InnateDB curated database and analysis tools (71), and network images were created with cytoscape (72, 73). Transcription factor binding site (based on Genome Browser position weight matrixes [PWMs]) and Gene Ontology biological process enrichment was performed with Enrichr (41). Ranking of significant processes was determined by sorting on the combined score and then sorting on the adjusted *P* value.

### Statistical analysis.

An unpaired *t* test was used to determine significant differences between the groups indicated in each figure for qRT-PCR analyses and ELISAs. Virus titers were analyzed by Mann-Whitney U test to assess the significance of differences between genotypes at each time. All quantifications are displayed as the mean ± standard deviation and were analyzed with Prism software (Prism 7; GraphPad, La Jolla, CA).

### Accession number(s).

Microarray data obtained in this study have been deposited in the NCBI Gene Expression Omnibus under GEO Series accession number GSE109069 in accordance with Minimum Information About & Microarray Experiment (MIAME) standards.

## References

[1] Dinarello CA 2009 Immunological and inflammatory functions of the interleukin-1 family. Annu Rev Immunol 27:519–550. doi:10.1146/annurev.immunol.021908.132612. 19302047

[2] Sen GC 2001 Viruses and interferons. Annu Rev Microbiol 55:255–281. doi:10.1146/annurev.micro.55.1.255. 11544356

[3] Sims JE, Smith DE 2010 The IL-1 family: regulators of immunity. Nat Rev Immunol 10:89–102. doi:10.1038/nri2691. 20081871

[4] Stetson DB, Medzhitov R 2006 Type I interferons in host defense. Immunity 25:373–381. doi:10.1016/j.immuni.2006.08.007. 16979569

[5] Mayer-Barber KD, Yan B 2017 Clash of the cytokine titans: counter-regulation of interleukin-1 and type I interferon-mediated inflammatory responses. Cell Mol Immunol 14:22–35. doi:10.1038/cmi.2016.25. 27264686 PMC5214938

[6] Durrant DM, Robinette ML, Klein RS 2013 IL-1R1 is required for dendritic cell-mediated T cell reactivation within the CNS during West Nile virus encephalitis. J Exp Med 210:503–516. doi:10.1084/jem.20121897. 23460727 PMC3600909

[7] Lazear HM, Pinto AK, Vogt MR, Gale M Jr., Diamond MS 2011 Beta interferon controls West Nile virus infection and pathogenesis in mice. J Virol 85:7186–7194. doi:10.1128/JVI.00396-11. 21543483 PMC3126609

[8] Ramos HJ, Lanteri MC, Blahnik G, Negash A, Suthar MS, Brassil MM, Sodhi K, Treuting PM, Busch MP, Norris PJ, Gale M Jr 2012 IL-1beta signaling promotes CNS-intrinsic immune control of West Nile virus infection. PLoS Pathog 8:e1003039. doi:10.1371/journal.ppat.1003039. 23209411 PMC3510243

[9] Suthar MS, Ma DY, Thomas S, Lund JM, Zhang N, Daffis S, Rudensky AY, Bevan MJ, Clark EA, Kaja MK, Diamond MS, Gale M Jr 2010 IPS-1 is essential for the control of West Nile virus infection and immunity. PLoS Pathog 6:e1000757. doi:10.1371/journal.ppat.1000757. 20140199 PMC2816698

[10] Gubler DJ 2007 The continuing spread of West Nile virus in the Western Hemisphere. Clin Infect Dis 45:1039–1046. doi:10.1086/521911. 17879923

[11] Krow-Lucal E, Lindsey NP, Lehman J, Fischer M, Staples JE 2017 West Nile virus and other nationally notifiable arboviral diseases—United States, 2015. MMWR Morb Mortal Wkly Rep 66:51–55. doi:10.15585/mmwr.mm6602a3. 28103209 PMC5657660

[12] Hayes EB, Komar N, Nasci RS, Montgomery SP, O’Leary DR, Campbell GL 2005 Epidemiology and transmission dynamics of West Nile virus disease. Emerg Infect Dis 11:1167–1173. doi:10.3201/eid1108.050289a. 16102302 PMC3320478

[13] Suthar MS, Diamond MS, Gale M Jr 2013 West Nile virus infection and immunity. Nat Rev Microbiol 11:115–128. doi:10.1038/nrmicro2950. 23321534

[14] Samuel MA, Diamond MS 2006 Pathogenesis of West Nile virus infection: a balance between virulence, innate and adaptive immunity, and viral evasion. J Virol 80:9349–9360. doi:10.1128/JVI.01122-06. 16973541 PMC1617273

[15] Davis LE, DeBiasi R, Goade DE, Haaland KY, Harrington JA, Harnar JB, Pergam SA, King MK, DeMasters BK, Tyler KL 2006 West Nile virus neuroinvasive disease. Ann Neurol 60:286–300. doi:10.1002/ana.20959. 16983682

[16] Sejvar JJ, Haddad MB, Tierney BC, Campbell GL, Marfin AA, Van Gerpen JA, Fleischauer A, Leis AA, Stokic DS, Petersen LR 2003 Neurologic manifestations and outcome of West Nile virus infection. JAMA 290:511–515. doi:10.1001/jama.290.4.511. 12876094

[17] Basu A, Krady JK, Levison SW 2004 Interleukin-1: a master regulator of neuroinflammation. J Neurosci Res 78:151–156. doi:10.1002/jnr.20266. 15378607

[18] Dinarello CA 2009 Interleukin-1beta and the autoinflammatory diseases. N Engl J Med 360:2467–2470. doi:10.1056/NEJMe0811014. 19494224

[19] Shrestha B, Zhang B, Purtha WE, Klein RS, Diamond MS 2008 Tumor necrosis factor alpha protects against lethal West Nile virus infection by promoting trafficking of mononuclear leukocytes into the central nervous system. J Virol 82:8956–8964. doi:10.1128/JVI.01118-08. 18632856 PMC2546880

[20] Akira S, Takeda K, Kaisho T 2001 Toll-like receptors: critical proteins linking innate and acquired immunity. Nat Immunol 2:675–680. doi:10.1038/90609. 11477402

[21] Wilkins C, Gale M Jr 2010 Recognition of viruses by cytoplasmic sensors. Curr Opin Immunol 22:41–47. doi:10.1016/j.coi.2009.12.003. 20061127 PMC3172156

[22] Lazear HM, Lancaster A, Wilkins C, Suthar MS, Huang A, Vick SC, Clepper L, Thackray L, Brassil MM, Virgin HW, Nikolich-Zugich J, Moses AV, Gale M Jr., Früh K, Diamond MS 2013 IRF-3, IRF-5, and IRF-7 coordinately regulate the type I IFN response in myeloid dendritic cells downstream of MAVS signaling. PLoS Pathog 9:e1003118. doi:10.1371/journal.ppat.1003118. 23300459 PMC3536698

[23] Mamane Y, Heylbroeck C, Génin P, Algarté M, Servant MJ, LePage C, DeLuca C, Kwon H, Lin R, Hiscott J 1999 Interferon regulatory factors: the next generation. Gene 237:1–14. doi:10.1016/S0378-1119(99)00262-0. 10524230

[24] Errett JS, Suthar MS, McMillan A, Diamond MS, Gale M Jr 2013 The essential, nonredundant roles of RIG-I and MDA5 in detecting and controlling West Nile virus infection. J Virol 87:11416–11425. doi:10.1128/JVI.01488-13. 23966395 PMC3807316

[25] Fredericksen BL, Keller BC, Fornek J, Katze MG, Gale M Jr 2008 Establishment and maintenance of the innate antiviral response to West Nile virus involves both RIG-I and MDA5 signaling through IPS-1. J Virol 82:609–616. doi:10.1128/JVI.01305-07. 17977974 PMC2224571

[26] Dinarello CA 1996 Biologic basis for interleukin-1 in disease. Blood 87:2095–2147. 8630372

[27] Ben-Sasson SZ, Caucheteux S, Crank M, Hu-Li J, Paul WE 2011 IL-1 acts on T cells to enhance the magnitude of in vivo immune responses. Cytokine 56:122–125. doi:10.1016/j.cyto.2011.07.006. 21843950 PMC3171626

[28] Kanneganti TD 2010 Central roles of NLRs and inflammasomes in viral infection. Nat Rev Immunol 10:688–698. doi:10.1038/nri2851. 20847744 PMC3909537

[29] Chang DM, Shaio MF 1994 Production of interleukin-1 (IL-1) and IL-1 inhibitor by human monocytes exposed to dengue virus. J Infect Dis 170:811–817. doi:10.1093/infdis/170.4.811. 7930722

[30] Negash AA, Ramos HJ, Crochet N, Lau DT, Doehle B, Papic N, Delker DA, Jo J, Bertoletti A, Hagedorn CH, Gale M Jr 2013 IL-1beta production through the NLRP3 inflammasome by hepatic macrophages links hepatitis C virus infection with liver inflammation and disease. PLoS Pathog 9:e1003330. doi:10.1371/journal.ppat.1003330. 23633957 PMC3635973

[31] Das S, Mishra MK, Ghosh J, Basu A 2008 Japanese encephalitis virus infection induces IL-18 and IL-1beta in microglia and astrocytes: correlation with in vitro cytokine responsiveness of glial cells and subsequent neuronal death. J Neuroimmunol 195:60–72. doi:10.1016/j.jneuroim.2008.01.009. 18374991

[32] King NJ, Getts DR, Getts MT, Rana S, Shrestha B, Kesson AM 2007 Immunopathology of flavivirus infections. Immunol Cell Biol 85:33–42. doi:10.1038/sj.icb.7100012. 17146465

[33] Guarda G, Braun M, Staehli F, Tardivel A, Mattmann C, Förster I, Farlik M, Decker T, Du Pasquier RA, Romero P, Tschopp J 2011 Type I interferon inhibits interleukin-1 production and inflammasome activation. Immunity 34:213–223. doi:10.1016/j.immuni.2011.02.006. 21349431

[34] Hu X, Ho HH, Lou O, Hidaka C, Ivashkiv LB 2005 Homeostatic role of interferons conferred by inhibition of IL-1-mediated inflammation and tissue destruction. J Immunol 175:131–138. doi:10.4049/jimmunol.175.1.131. 15972639

[35] Mayer-Barber KD, Andrade BB, Barber DL, Hieny S, Feng CG, Caspar P, Oland S, Gordon S, Sher A 2011 Innate and adaptive interferons suppress IL-1alpha and IL-1beta production by distinct pulmonary myeloid subsets during Mycobacterium tuberculosis infection. Immunity 35:1023–1034. doi:10.1016/j.immuni.2011.12.002. 22195750 PMC3246221

[36] Tarassishin L, Suh HS, Lee SC 2011 Interferon regulatory factor 3 plays an anti-inflammatory role in microglia by activating the PI3K/Akt pathway. J Neuroinflamm 8:187. doi:10.1186/1742-2094-8-187. PMC3259120 22208359

[37] Hisaeda K, Inokuchi A, Nakamura T, Iwamoto Y, Kohno K, Kuwano M, Uchiumi T 2004 Interleukin-1beta represses MRP2 gene expression through inactivation of interferon regulatory factor 3 in HepG2 cells. Hepatology 39:1574–1582. doi:10.1002/hep.20216. 15185298

[38] Mayer-Barber KD, Andrade BB, Oland SD, Amaral EP, Barber DL, Gonzales J, Derrick SC, Shi R, Kumar NP, Wei W, Yuan X, Zhang G, Cai Y, Babu S, Catalfamo M, Salazar AM, Via LE, Barry CE III, Sher A 2014 Host-directed therapy of tuberculosis based on interleukin-1 and type I interferon crosstalk. Nature 511:99–103. doi:10.1038/nature13489. 24990750 PMC4809146

[39] Goritzka M, Durant LR, Pereira C, Salek-Ardakani S, Openshaw PJ, Johansson C 2014 Alpha/beta interferon receptor signaling amplifies early proinflammatory cytokine production in the lung during respiratory syncytial virus infection. J Virol 88:6128–6136. doi:10.1128/JVI.00333-14. 24648449 PMC4093897

[40] Ichikawa T, Nakao K, Nakata K, Yamashita M, Hamasaki K, Shigeno M, Abiru S, Ishikawa H, Ishii N, Eguchi K 2002 Involvement of IL-1beta and IL-10 in IFN-alpha-mediated antiviral gene induction in human hepatoma cells. Biochem Biophys Res Commun 294:414–422. doi:10.1016/S0006-291X(02)00502-8. 12051728

[41] Chen EY, Tan CM, Kou Y, Duan Q, Wang Z, Meirelles GV, Clark NR, Ma’ayan A 2013 Enrichr: interactive and collaborative HTML5 gene list enrichment analysis tool. BMC Bioinformatics 14:128. doi:10.1186/1471-2105-14-128. 23586463 PMC3637064

[42] Grandvaux N, Servant MJ, tenOever B, Sen GC, Balachandran S, Barber GN, Lin R, Hiscott J 2002 Transcriptional profiling of interferon regulatory factor 3 target genes: direct involvement in the regulation of interferon-stimulated genes. J Virol 76:5532–5539. doi:10.1128/JVI.76.11.5532-5539.2002. 11991981 PMC137057

[43] Guo J, Peters KL, Sen GC 2000 Induction of the human protein P56 by interferon, double-stranded RNA, or virus infection. Virology 267:209–219. doi:10.1006/viro.1999.0135. 10662616

[44] Lazear HM, Daniels BP, Pinto AK, Huang AC, Vick SC, Doyle SE, Gale M Jr., Klein RS, Diamond MS 2015 Interferon-lambda restricts West Nile virus neuroinvasion by tightening the blood-brain barrier. Sci Transl Med 7:284ra59. doi:10.1126/scitranslmed.aaa4304. PMC4435724 25904743

[45] DiDonato JA, Hayakawa M, Rothwarf DM, Zandi E, Karin M 1997 A cytokine-responsive IkappaB kinase that activates the transcription factor NF-kappaB. Nature 388:548–554. doi:10.1038/41493. 9252186

[46] Fitzgerald KA, McWhirter SM, Faia KL, Rowe DC, Latz E, Golenbock DT, Coyle AJ, Liao SM, Maniatis T 2003 IKKepsilon and TBK1 are essential components of the IRF3 signaling pathway. Nat Immunol 4:491–496. doi:10.1038/ni921. 12692549

[47] Sharma S, tenOever BR, Grandvaux N, Zhou GP, Lin R, Hiscott J 2003 Triggering the interferon antiviral response through an IKK-related pathway. Science 300:1148–1151. doi:10.1126/science.1081315. 12702806

[48] Perwitasari O, Cho H, Diamond MS, Gale M Jr 2011 Inhibitor of κB kinase epsilon (IKK(epsilon)), STAT1, and IFIT2 proteins define novel innate immune effector pathway against West Nile virus infection. J Biol Chem 286:44412–44423. doi:10.1074/jbc.M111.285205. 22065572 PMC3247963

[49] tenOever BR, Ng SL, Chua MA, McWhirter SM, García-Sastre A, Maniatis T 2007 Multiple functions of the IKK-related kinase IKKepsilon in interferon-mediated antiviral immunity. Science 315:1274–1278. doi:10.1126/science.1136567. 17332413

[50] Podolin PL, Callahan JF, Bolognese BJ, Li YH, Carlson K, Davis TG, Mellor GW, Evans C, Roshak AK 2005 Attenuation of murine collagen-induced arthritis by a novel, potent, selective small molecule inhibitor of IκB kinase 2, TPCA-1 (2-[(aminocarbonyl)amino]-5-(4-fluorophenyl)-3-thiophenecarboxamide), occurs via reduction of proinflammatory cytokines and antigen-induced T cell proliferation. J Pharmacol Exp Ther 312:373–381. doi:10.1124/jpet.104.074484. 15316093

[51] Clark K, Peggie M, Plater L, Sorcek RJ, Young ER, Madwed JB, Hough J, McIver EG, Cohen P 2011 Novel cross-talk within the IKK family controls innate immunity. Biochem J 434:93–104. doi:10.1042/BJ20101701. 21138416

[52] Thanos D, Maniatis T 1992 The high mobility group protein HMG I(Y) is required for NF-kappa B-dependent virus induction of the human IFN-beta gene. Cell 71:777–789. doi:10.1016/0092-8674(92)90554-P. 1330326

[53] Honda K, Yanai H, Negishi H, Asagiri M, Sato M, Mizutani T, Shimada N, Ohba Y, Takaoka A, Yoshida N, Taniguchi T 2005 IRF-7 is the master regulator of type-I interferon-dependent immune responses. Nature 434:772–777. doi:10.1038/nature03464. 15800576

[54] Couper KN, Blount DG, Riley EM 2008 IL-10: the master regulator of immunity to infection. J Immunol 180:5771–5777. doi:10.4049/jimmunol.180.9.5771. 18424693

[55] Wong PK, Egan PJ, Croker BA, O’Donnell KS, Sims NA, Drake S, Kiu H, McManus EJ, Alexander WS, Roberts AW, Wicks IP 2006 SOCS-3 negatively regulates innate and adaptive immune mechanisms in acute IL-1-dependent inflammatory arthritis. J Clin Invest 116:1571–1581. doi:10.1172/JCI25660. 16710471 PMC1462939

[56] Ivashkiv LB, Donlin LT 2014 Regulation of type I interferon responses. Nat Rev Immunol 14:36–49. doi:10.1038/nri3581. 24362405 PMC4084561

[57] López de Padilla CM, Niewold TB 2016 The type I interferons: basic concepts and clinical relevance in immune-mediated inflammatory diseases. Gene 576:14–21. doi:10.1016/j.gene.2015.09.058. 26410416 PMC4666791

[58] Novikov A, Cardone M, Thompson R, Shenderov K, Kirschman KD, Mayer-Barber KD, Myers TG, Rabin RL, Trinchieri G, Sher A, Feng CG 2011 Mycobacterium tuberculosis triggers host type I IFN signaling to regulate IL-1beta production in human macrophages. J Immunol 187:2540–2547. doi:10.4049/jimmunol.1100926. 21784976 PMC3159798

[59] Redford PS, Mayer-Barber KD, McNab FW, Stavropoulos E, Wack A, Sher A, O’Garra A 2014 Influenza A virus impairs control of Mycobacterium tuberculosis coinfection through a type I interferon receptor-dependent pathway. J Infect Dis 209:270–274. doi:10.1093/infdis/jit424. 23935205 PMC3873785

[60] Coulombe F, Jaworska J, Verway M, Tzelepis F, Massoud A, Gillard J, Wong G, Kobinger G, Xing Z, Couture C, Joubert P, Fritz JH, Powell WS, Divangahi M 2014 Targeted prostaglandin E2 inhibition enhances antiviral immunity through induction of type I interferon and apoptosis in macrophages. Immunity 40:554–568. doi:10.1016/j.immuni.2014.02.013. 24726877

[61] Rivieccio MA, John GR, Song X, Suh HS, Zhao Y, Lee SC, Brosnan CF 2005 The cytokine IL-1beta activates IFN response factor 3 in human fetal astrocytes in culture. J Immunol 174:3719–3726. doi:10.4049/jimmunol.174.6.3719. 15749911

[62] González-Navajas JM, Law J, Nguyen KP, Bhargava M, Corr MP, Varki N, Eckmann L, Hoffman HM, Lee J, Raz E 2010 Interleukin 1 receptor signaling regulates DUBA expression and facilitates Toll-like receptor 9-driven antiinflammatory cytokine production. J Exp Med 207:2799–2807. doi:10.1084/jem.20101326. 21115691 PMC3005235

[63] Wienerroither S, Shukla P, Farlik M, Majoros A, Stych B, Vogl C, Cheon H, Stark GR, Strobl B, Müller M, Decker T 2015 Cooperative transcriptional activation of antimicrobial genes by STAT and NF-κB pathways by concerted recruitment of the mediator complex. Cell Rep 12:300–312. doi:10.1016/j.celrep.2015.06.021. 26146080 PMC4521078

[64] Fujita T, Reis LF, Watanabe N, Kimura Y, Taniguchi T, Vilcek J 1989 Induction of the transcription factor IRF-1 and interferon-beta mRNAs by cytokines and activators of second-messenger pathways. Proc Natl Acad Sci U S A 86:9936–9940. doi:10.1073/pnas.86.24.9936. 2557635 PMC298617

[65] Häcker H, Redecke V, Blagoev B, Kratchmarova I, Hsu LC, Wang GG, Kamps MP, Raz E, Wagner H, Häcker G, Mann M, Karin M 2006 Specificity in Toll-like receptor signalling through distinct effector functions of TRAF3 and TRAF6. Nature 439:204–207. doi:10.1038/nature04369. 16306937

[66] Tseng PH, Matsuzawa A, Zhang W, Mino T, Vignali DA, Karin M 2010 Different modes of ubiquitination of the adaptor TRAF3 selectively activate the expression of type I interferons and proinflammatory cytokines. Nat Immunol 11:70–75. doi:10.1038/ni.1819. 19898473 PMC2872790

[67] Suthar MS, Brassil MM, Blahnik G, McMillan A, Ramos HJ, Proll SC, Belisle SE, Katze MG, Gale M Jr 2013 A systems biology approach reveals that tissue tropism to West Nile virus is regulated by antiviral genes and innate immune cellular processes. PLoS Pathog 9:e1003168. doi:10.1371/journal.ppat.1003168. 23544010 PMC3567171

[68] Gentleman RC, Carey VJ, Bates DM, Bolstad B, Dettling M, Dudoit S, Ellis B, Gautier L, Ge Y, Gentry J, Hornik K, Hothorn T, Huber W, Iacus S, Irizarry R, Leisch F, Li C, Maechler M, Rossini AJ, Sawitzki G, Smith C, Smyth G, Tierney L, Yang JY, Zhang J 2004 Bioconductor: open software development for computational biology and bioinformatics. Genome Biol 5:R80. doi:10.1186/gb-2004-5-10-r80. 15461798 PMC545600

[69] R Development Core Team 2012 R: a language and environment for statistical computing. R Foundation for Statistical Computing, Vienna, Austria https://www.r-project.org/.

[70] Smyth GK 2004 Linear models and empirical Bayes methods for assessing differential expression in microarray experiments. Stat Appl Genet Mol Biol 3:Article3. doi:10.2202/1544-6115.1027. 16646809

[71] Lynn DJ, Chan C, Naseer M, Yau M, Lo R, Sribnaia A, Ring G, Que J, Wee K, Winsor GL, Laird MR, Breuer K, Foroushani AK, Brinkman FS, Hancock RE 2010 Curating the innate immunity interactome. BMC Syst Biol 4:117. doi:10.1186/1752-0509-4-117. 20727158 PMC2936296

[72] Cline MS, Smoot M, Cerami E, Kuchinsky A, Landys N, Workman C, Christmas R, Avila-Campilo I, Creech M, Gross B, Hanspers K, Isserlin R, Kelley R, Killcoyne S, Lotia S, Maere S, Morris J, Ono K, Pavlovic V, Pico AR, Vailaya A, Wang PL, Adler A, Conklin BR, Hood L, Kuiper M, Sander C, Schmulevich I, Schwikowski B, Warner GJ, Ideker T, Bader GD 2007 Integration of biological networks and gene expression data using cytoscape. Nat Protoc 2:2366–2382. doi:10.1038/nprot.2007.324. 17947979 PMC3685583

[73] Shannon P, Markiel A, Ozier O, Baliga NS, Wang JT, Ramage D, Amin N, Schwikowski B, Ideker T 2003 Cytoscape: a software environment for integrated models of biomolecular interaction networks. Genome Res 13:2498–2504. doi:10.1101/gr.1239303. 14597658 PMC403769

[74] Kent WJ, Sugnet CW, Furey TS, Roskin KM, Pringle TH, Zahler AM, Haussler D 2002 The human genome browser at UCSC. Genome Res 12:996–1006. doi:10.1101/gr.229102. 12045153 PMC186604

