# Supplementary figures and images for: Interleukin-1β Signaling in Dendritic Cells Induces Antiviral Interferon Responses
Source: mBio. 2018 Mar 20;9(2):e00342-18. doi: 10.1128/mBio.00342-18 (PMC5874908; doi:10.1128/mBio.00342-18)

Figure S1. IL-1R signaling is necessary for induction of antiviral response genes

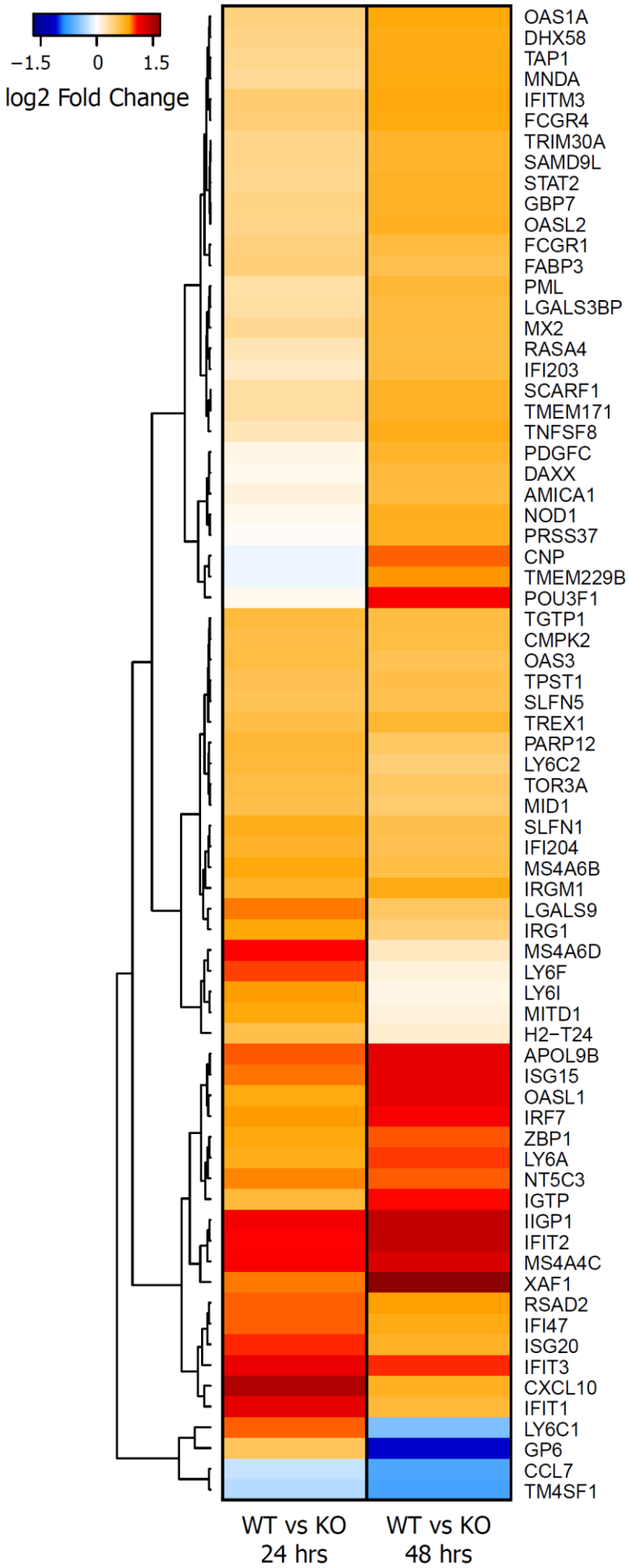

Supplement: FIG S1 [file mbo002183791sf1.pdf]

Figure S2. IL-1 $\beta$  treatment drives inflammatory response genes

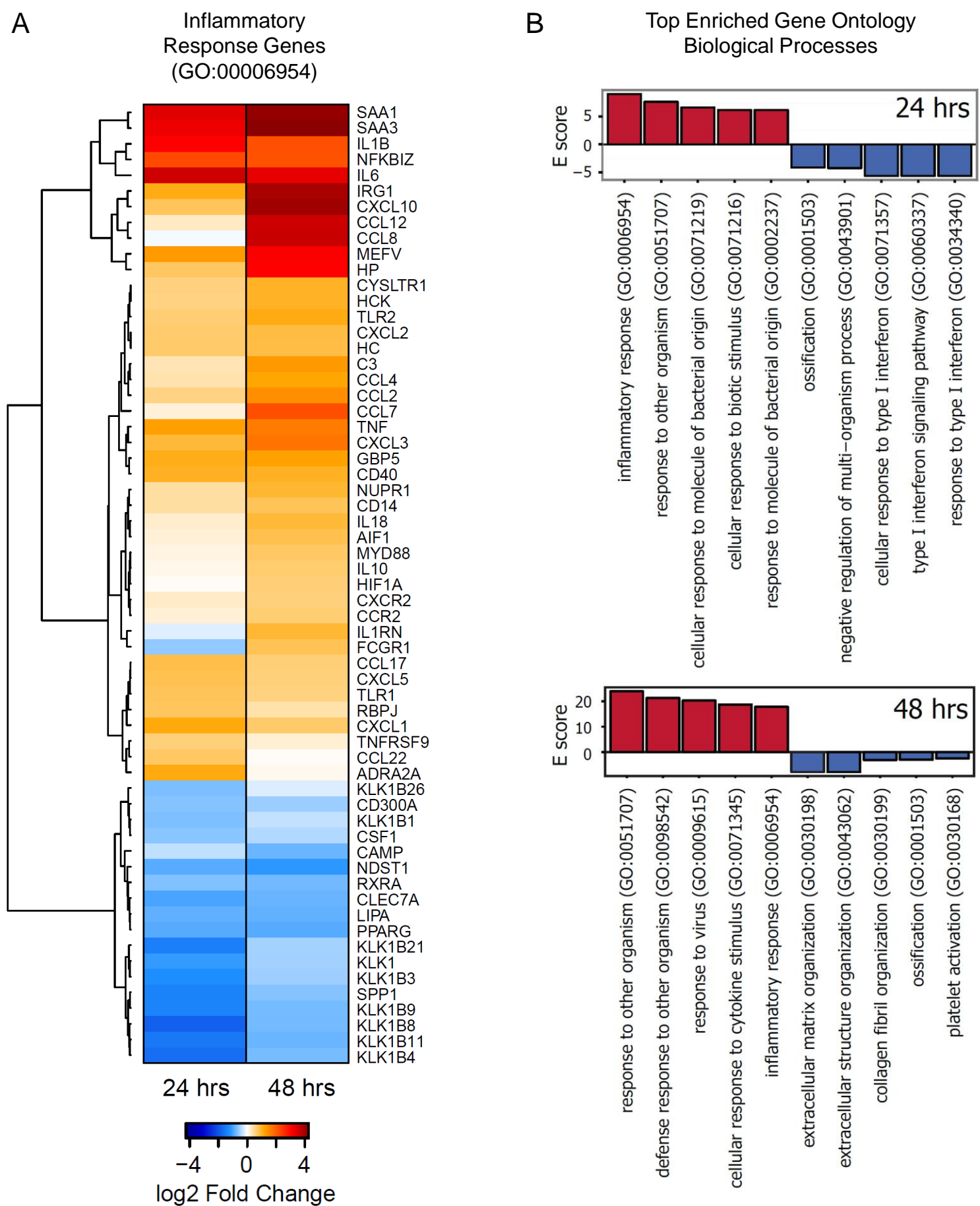

Supplement: FIG S2 [file mbo002183791sf2.pdf]

Figure S3. IL-1 $\beta$  treatment drives expression of ISGs

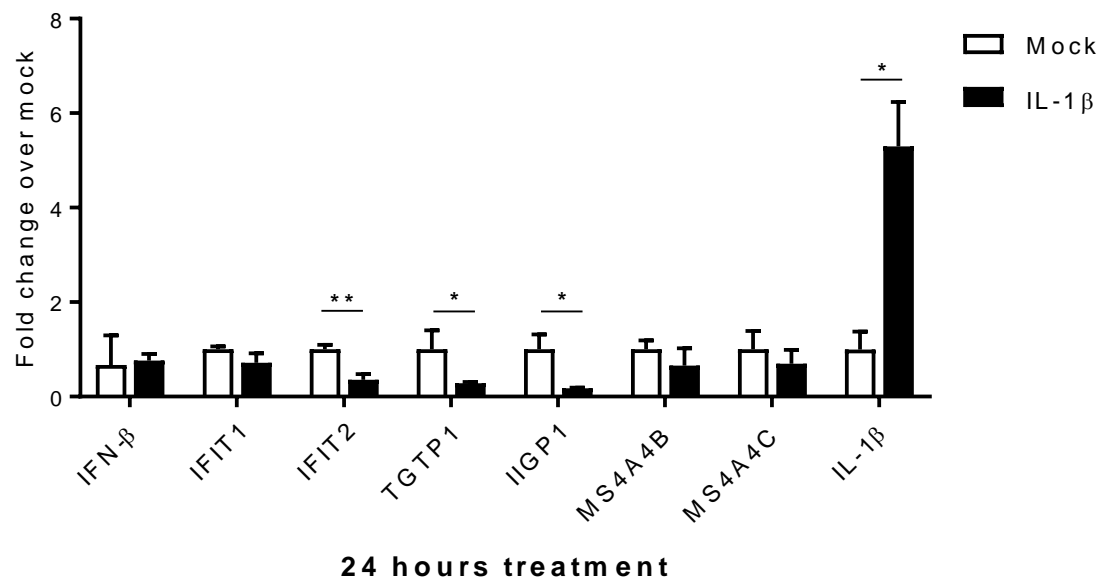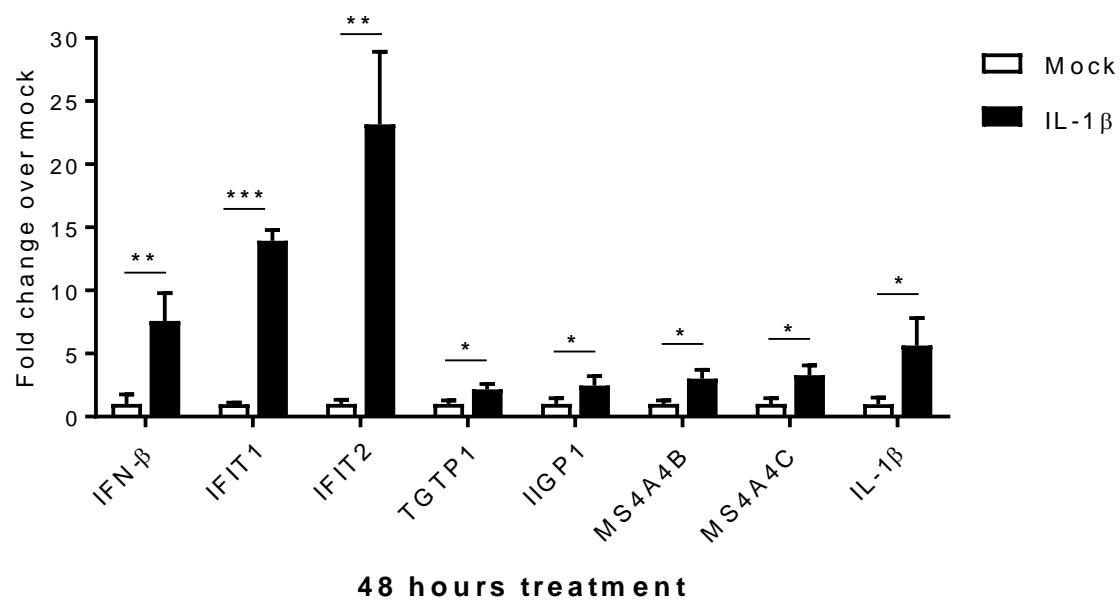

Supplement: FIG S3 [file mbo002183791sf3.pdf]
